# Effect of Autolyzed *Yarrowia lipolytica* on the Growth Performance, Antioxidant Capacity, Intestinal Histology, Microbiota, and Transcriptome Profile of Juvenile Largemouth Bass (*Micropterus salmoides*)

**DOI:** 10.3390/ijms231810780

**Published:** 2022-09-15

**Authors:** Hui Fei, Yan Cheng, Huimin Zhang, Xiang Yu, Shunfa Yi, Mengmeng Huang, Shun Yang

**Affiliations:** 1 College of Life Sciences and Medicine, Zhejiang Sci-Tech University, Hangzhou 310018, China; 2 Zhejiang Provincial Key Laboratory of Silkworm Bioreactor and Biomedicine, Zhejiang Sci-Tech University, Hangzhou 310018, China; 3 Zhejiang Development &Planning Institute, Hangzhou 310012, China

**Keywords:** *Micropterus salmoides*, *Yarrowia lipolytica*, transcriptome, intestinal microbiota, antioxidant capacity

## Abstract

The improper components of formulated feed can cause the intestinal dysbiosis of juvenile largemouth bass and further affect fish health. A 28 day feeding trial was conducted to investigate the effect of partially replacing fish meal (FM) with autolyzed *Yarrowia lipolytica* (YL) on juvenile largemouth bass (*Micropterus salmoides*). We considered four diets—control, YL25, YL50, and YL75—in which 0%, 25%, 50%, and 75% of the FM content, respectively, was replaced with YL. According to results, the weight gain rate (WGR) and specific growth rate (SGR) of the fish with the YL25 and YL50 diets were significantly higher than the WGR and SGR with the control diet, while the YL75 diet significantly reduced fish growth and antioxidant enzymes activities, and shortened the villus height in the intestinal mucosa. The 16S rRNA analysis of the intestinal microbiota showed that the relative abundance of *Mycoplasma* was significantly increased with the YL25 and YL50 diets, while the *Enterobacteriacea* content was increased with the YL75 diet. Moreover, our transcriptome analysis revealed that certain differentially expressed genes (DEGs) that are associated with growth, metabolism, and immunity were modulated by YL inclusion treatment. Dietary YL25 and YL50 significantly reduced the mRNA level of ERBB receptor feedback inhibitor 1 (*errfi1*) and dual-specificity phosphatases (*dusp*), while the expression of the suppressor of cytokine signaling 1 (*socs1*), the transporter associated with antigen processing 2 subunit type a (*tap2a*), and the major histocompatibility complex class I-related gene (*MHC-I-l*) were sharply increased with YL75 treatment. We determined that the optimum dose of dietary YL required for maximum growth without any adverse influence on intestinal health was 189.82 g/kg (with 31.63% of the fishmeal replaced by YL), while an excessive substitution of YL for fishmeal led to suppressed growth and antioxidant capacity, as well as intestinal damage for juvenile largemouth bass.

## 1. Introduction

The largemouth bass (*Micropterus salmoides*) is an important freshwater fish species with great economic value in aquaculture, as it is highly preferred by consumers [1,2]. The frequent outbreaks of diseases caused by various pathogens, such as *Aeromonas hydrophila*, *Nocardia seriolae*, and *Micropterus salmoides* rhabdovirus (MSRV), have become a limiting factor in the development of largemouth bass farming [3,4]. In addition, the largemouth bass is a fierce carnivorous fish and juvenile largemouth bass practice cannibalism, which significantly reduces the production of juvenile largemouth bass [5]. 

More importantly, rising prices and supply shortages of fishmeal (FM) seriously restrict the development of aquaculture. In recent decades, an enormous amount of research has focused on the macronutrient requirements of largemouth bass [6,7,8,9]. Nevertheless, improper components of formulated feed can cause a nutrition metabolism disturbance and damage to the intestinal health of largemouth bass [8,9,10]. For example, various plant-based ingredients, such as soybean meal, rapeseed meal, and cottonseed, have been used as FM alternatives in aquafeed, due to their stable yields and low costs. Nevertheless, plant-based proteins naturally contain anti-nutritional factors, and their unbalanced amino acid composition and poor palatability impair the growth and health of aquatic animals [7,9,11,12,13]. Therefore, it is necessary to develop cost-effective and healthy FM-substitution ingredients for the sustainable development of largemouth bass.

*Yarrowia lipolytica* (YL) is a yeast species that contains high crude protein, complete amino acid components, a high utilization rate, vitamins, inorganic salt, fat, and sugar. It not only contains a high-quality single-cell protein (SCP), but it also has been proven to display a probiotic effect [10,14]. Furthermore, YL could be fermented on a large scale by using industrial glycerin as a substrate, and it could produce SCP that contains high levels of lysine, serine, and proline [15]. In addition, compared with other yeast species, YL can accumulate high levels of lipids by both de novo synthesis and uptake from exogenous sources [16,17], especially polyunsaturated fatty acid (PUFA), which can enhance the immune capacity of an organism [18]. 

In the aquaculture field, when fed to Atlantic salmon, dietary YL biomass increases the availability of n-3 highly unsaturated fatty acids [19]. Moreover, increasing evidence has demonstrated the beneficial effects of YL on the antioxidant capacity and the immune-related gene expression of zebrafish (*Danio rerio*) [10], pacific red snapper (*Lutjanus peru*) [20] and shrimp (*Litopenaeus vannamei*) [21], which indicates the potential application of *Y. lipolytica*-based immunostimulants for aquatic animals. Nevertheless, little information is currently available on the influence of dietary YL on juvenile largemouth bass; therefore, this influence deserves an in-depth investigation.

In recent years, growing evidence revealed that dietary FM substitution affects the intestinal microbiome in most fish species [9,22], and the influence of intestinal microbes on the physiological performance of a host has attracted extensive attention [23,24]. For instance, Yang et al. demonstrated that the proper inclusion of fermented soybean meal promoted the diversity of the Firmicutes in the largemouth bass intestine, which could help in the digestion of cellulose when fish are fed diets with a high inclusion of plant protein [9]. Similarly, dietary multi-strain yeast fractions increased the abundance of *Cetobacterium* in the largemouth bass intestine, which may improve growth performance and gut health in the fish [8]. Therefore, the effect of dietary YL on the development of the intestine in juvenile fish may be partly related to variations in intestinal microbiota.

In addition, a transcriptome sequencing was applied to understand how dietary changes affected the physiological processes of fish [25,26]. Lu et al. investigate the growth performance and liver transcriptome responses of juvenile *Erythroculter ilishaeformis* that were fed with different levels of dietary protein, and provided a comprehensive understanding of the feed formulation and molecular mechanism that underlie the effects of dietary protein on *E. ilishaeformis* [25]. Similarly, a transcriptome analysis revealed that dietary daidzein improved the intestinal development of turbot, which might be related to the activation of the Wnt/β-catenin signaling pathway [26]. Accordingly, transcriptome is being applied in non-model animals, and helps in comprehensively understanding the molecular basis for phenotypic differences and in identifying the differentially expressed genes and pathways in certain tissues that are involved in the regulation of dietary components.

With this background we conducted a 28 day feeding trial to investigate the effects of dietary YL on the growth performance, antioxidant capacity, intestinal histology, microbiota, and transcriptome response of juvenile largemouth bass, with the aim of evaluating the feasibility of YL as a replacement ingredient in FM.

## 2. Results

### 2.1. Growth Performance

The growth performance of juvenile largemouth bass was determined over a period of 28 day feeding trial. The WGR and SGR of fish fed with the YL25 and YL50 diets were significantly higher than that of fish fed with the control diety (*p*_quadratic_ < 0.001). The quadratic polynomial test and regression relationships (y = −452.16x^2^ + 286.19x + 315.7, R² = 0.9294) indicated that the optimal dose of dietary YL substitution for maximum growth was 189.82 g/kg (with 31.63% of the fishmeal replaced by YL). The maximum condition factor (CF) was determined in the YL50 treatment, while the YL75 diet significantly decreased the CF (*p*_quadratic_ = 0.007) and increased the feed conversion ratio (FCR) of the juvenile largemouth bass (*p*_quadratic_ = 0.001) (Table 1).

### 2.2. Flesh Composition

The flesh composition of the largemouth bass at the end of the feeding trial is presented in Table 2. The inclusion of YL produced no significant influence on the moisture and crude protein content of the fish in a quadratic manner (*p*_quadratic_ > 0.05). Nevertheless, significant differences were observed in the lipid content of fish flesh in all four groups. The highest crude lipid content was observed in the YL50 treatment (*p*_quadratic_ < 0.001).

### 2.3. Antioxidant-Related Enzyme Activities

**Figure 1:**
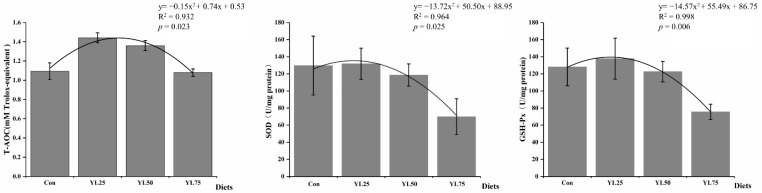
Effects of dietary YL on antioxidant-related enzyme activities of intestine in juvenile largemouth bass. T-AOC, total antioxidant capacity; SOD, superoxide dismutase; GSH-Px, glutathione peroxidase. Con was the control diet. In the other three diets, 25%, 50%, 75% of the fish meal was replaced with YL. The three diets were named, respectively, YL25, YL50, and YL75.

The intestine antioxidant-related enzyme activities are shown in Figure 1. The T-AOC, SOD, and GSH-Px activity showed a tendency to decrease, after an initial increase, with the increase in the YL inclusion level, and the lowest activity was observed in the YL75 treatment in a quadratic manner (T-AOC: *p*_quadratic_ = 0.023; SOD: *p*_quadratic_ = 0.025; GSH-Px: *p*_quadratic_ = 0.006). The quadratic polynomial test and the regression relationships indicated that the optimal doses of dietary YL substitution for the maximum activity of T-AOC, SOD, and GSH-Px were 220.2 g/kg (with 36.7% of the fishmeal replaced by YL), 126 g/kg (with 21.0% of the fishmeal replaced by YL), and 135.6 g/kg (with 22.6% of the fishmeal replaced by YL).

### 2.4. Intestinal Histomorphology

**Figure 2:**
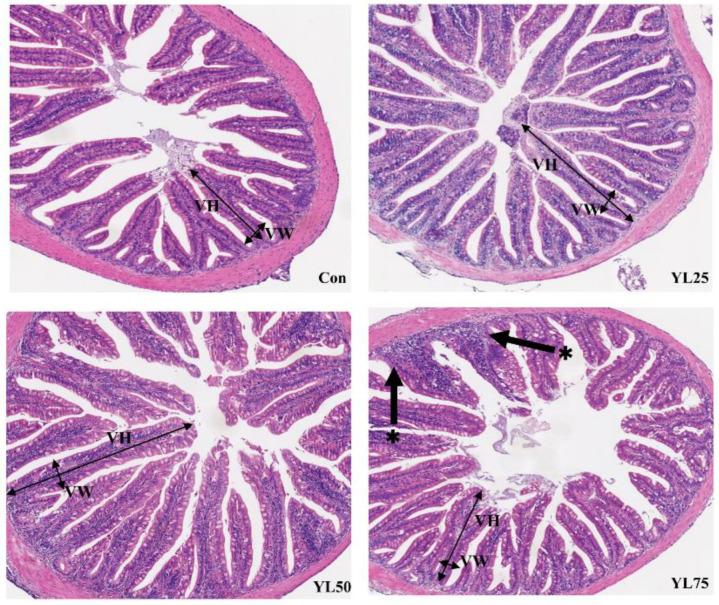
The mid-intestinal histology in largemouth bass fed different YL inclusion diets for 28 days. Magnification ×200. Black asterisks represent distorted crypt structure in intestine mucosa. Black thick arrows represent the villus height (VH) and villus width (VW). Con was the control diet. In the other three diets, 25%, 50%, 75% of the fish meal was replaced with YL. The three diets were named YL25, YL50, and YL75, respectively.

As shown in Figure 2, fish in the control, YL25, or YL50 treatments had intact intestinal structures, including neat gut villus, smooth intestinal mucosa, and no obervable inflammation or damage. However, when 75% of the dietary FM was replaced by YL, the intestinal villi of the fish appeared shorter and more sparse, with distorted crypt structure in the YL75 treatment. A statistical analysis showed that a significantly lower villus height was observed in the YL75 treatment in a quadratic manner (*p*_quadratic_ = 0.002) (Table 3).

### 2.5. Analysis of Intestinal Microbiota between Control and YL Treatments

**Figure 3:**
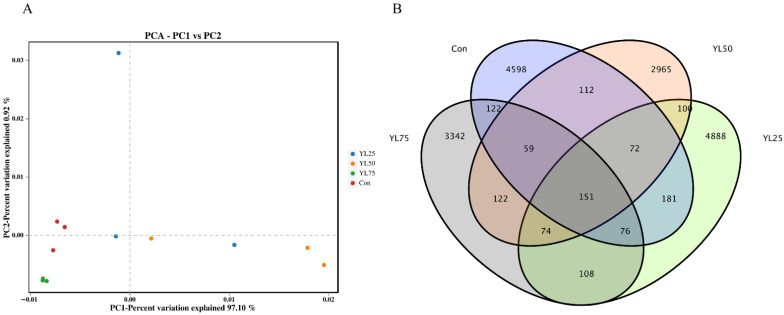
The PCA and Venn diagram of intestinal microbiota of largemouth bass fed with four experimental diets. (**A**) The principal component analysis of the control and YL treatments. Red splashes represent control treatment, blue splashes represent YL25 treatment, orange splashes represent YL50 treatment, green splashes represent YL75 treatment. (**B**) The Venn diagram showing out numbers. Con was the control diet. In the other three diets, 25%, 50%, 75% of the fish meal was replaced with YL. The diets were named YL25, YL50, and YL75, respectively.

The intestinal microbiota in the control and YL treatments were analyzed using the high-throughput sequencing of the 16S rRNA gene. A total of 959,962 raw reads and 958,739 clean reads were obtained from 12 samples (Table S1). To better understand the difference in microbial communities between the control and YL treatments, the Shannon, Simpson, and Chao1 indices were calculated from the OTUs (Table S2). The results revealed that the Shannon and Simpson indices of the intestinal microbiota were significantly affected in the YL50 inclusion treatment (*p* < 0.05). Moreover, the principal component analysis (PCA) showed that the intestinal microbiota of the control and YL treatments were grouped separately (Figure 3A).

**Figure 4:**
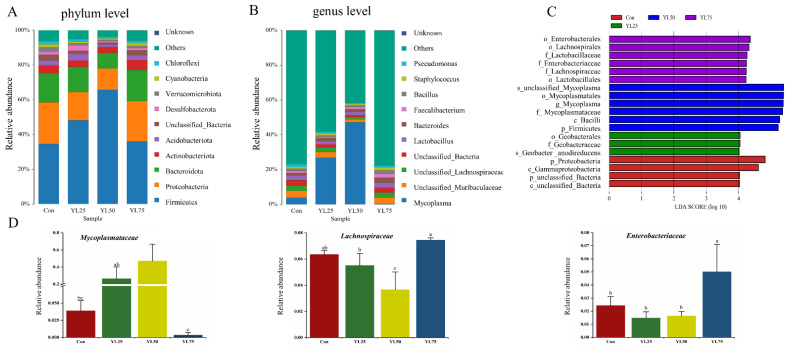
Difference analysis of intestinal microbiota between control and YL treatments of largemouth bass. (**A**) The bar plots for the microbial community at the phylum level. (**B**)The bar plots for the microbial community at the genus level. (**C**) The LDA scores of different microbes between the control YL treatments. Red means the control group; green means the YL25 treatment, blue means the YL50 treatment, purple means the YL75 treatment. (**D**) Comparison of the relative abundance of different microbes between the control and YL treatments. Values in rows with different superscript letters have significant differences (*p* < 0.05). Con was the control diet. In the other three diets, 25%, 50%, 75% of the fish meal was replaced with YL. The diets were named YL25, YL50, and YL75, respectively.

Additionally, the Venn diagram showed that the YL25 treatment had the largest number of intestinal microbial OTU, followed by the control, YL75, and YL50 treatments (Figure 3B). Microbial community bar plots at the phylum level revealed that Firmicutes were the dominant taxon in all four treatments, followed by Proteobacteria and Bacteroidetes (Figure 4A). At the genus level, unclassified_Muribaculaceae was the main species, followed by Mycoplasma and unclassified_Bacteria in the control treatment. Mycoplasma was the dominant taxon in the YL25 treatment, followed by unclassified_Muribaculaceae and unclassified_Lachnospiraceae. Similarly, Mycoplasma was the dominate species in the YL50 treatment, followed by Lactobacillus and unclassified_Muribaculaceae. In the YL75 treatment, unclassified_Muribaculaceae was the dominant taxon, followed by Bacteroides and unclassified_Lachnospiraceae (Figure 4B). Moreover, through the LEfSe analysis, we found the relative abundance of Enterobacteriaceae in the YL75 treatment, and that Mycoplasmataceae in the YL25 and YL5 treatments were significantly higher than in the control treatment at the family level, whereas Lachnospiraceae in the YL50 treatment was significantly lower than in the control treatment (*p* < 0.05) (Figure 4C,D).

### 2.6. Differential Gene Expression between the Control and YL Treatments

**Figure 5:**
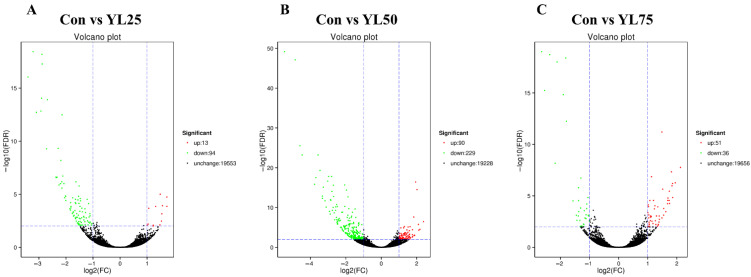
Volcano plot of differentially expressed genes in the YL25 treatment vs. the control treatment (**A**), the YL50 treatment vs. the control treatment (**B**), and the YL75 treatment vs. the control treatment (**C**). The x-axis shows the fold change in gene expression between the control treatment and the YL treatments, and the y-axis shows the statistical significance of the differences. Red splashes represent significantly upregulated genes. Green splashes represent significantly downregulated genes. Black splashes indicate no difference. Con was the control diet. In the other three diets, 25%, 50%, 75% of the fish meal was replaced with YL. The diets were named YL25, YL50, and YL75, respectively.

To analyze the effect of dietary YL on largemouth bass in the gene levels, intestines were collected after the feeding trial for transcriptome analysis. After sequencing, an average of 75.19 Gb clean reads were obtained for all 12 samples. The GC content was 47.97% and Q30 was 96.70%. The average mapping rate for all clean reads ranged from 80.65% to 95.09% (Table S3). Then, the DEGs were identified on the basis of expression-fold change ≥ 2 and FDR < 0.01. Compared with the control treatment, there were 13, 90, 51 upregulated genes and 94, 229, and 36 downregulated genes in the YL25, YL50, and YL75 treatments, respectively. The numbers of DEGs between the control and YL treatments are shown in Figure 5.

**Figure 6:**
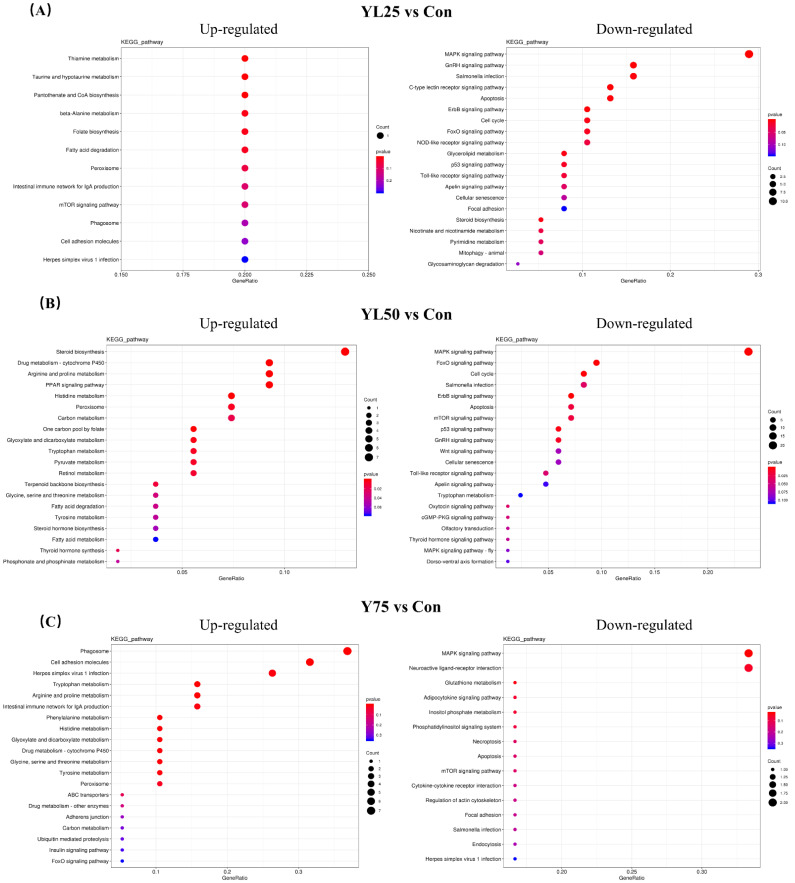
KEGG enrichment analysis of DEGs in transcriptome following dietary YL inclusion. (**A**) KEGG enrichment analysis of the regulated genes in the YL25 treatment. (**B**) KEGG enrichment analysis of the regulated genes in the YL50 treatment. (**C**) KEGG enrichment analysis of the regulated genes in the YL75 treatment. Con was the control diet. In the other three diets, 25%, 50%, 75% of the fish meal was replaced with YL. The diets were named YL25, YL50, and YL75, respectively.

The enrichment of DEGs between the control and the YL inclusion treatments (YL25 vs. Con, YL50 vs. Con, YL75 vs. Con) was analyzed by using the Encyclopedia of GO and KEGG bases for functional analysis. Genes were classified into three major functional categories, based on GO annotation (Figures S1–S3). KEGG enrichment analysis showed that DEGs enriched in the MAPK signaling pathway and the Gnrh signaling pathway were downregulated for the YL25 treatment when compared with the control treatment, while few genes were upregulated. For the YL50 treatment vs. the control treatment, the upregulated genes were primarily enriched in lipid and AA metabolic pathways, including steroid biosynthesis, the PPAR signaling pathway, arginine and proline metabolism, and histidine metabolism, while downregulated genes were mainly enriched in the MAPK signaling pathway and the FoxO signaling pathway. For the YL75 treatment vs. the control treatment, the upregulated genes were mainly enriched in phagosome and cell adhesion molecules, while few genes were downregulated (Figure 6). In addition, transcriptome analysis showed that the expression of certain genes associated with growth (*errfi1* and *socs1*), metabolism (*fasn*, *fabp2*, *gadl1*, *gldc*, *ftcd*, and *mao*) and immunity (*dusp1*, *dusp5*, *tap2a*, and *MHC-I-l*), which play important roles in growth and immunity, were modulated by dietary YL inclusion (Table S4).

### 2.7. Validation of Selected DEGs by qRT-PCR

**Figure 7:**
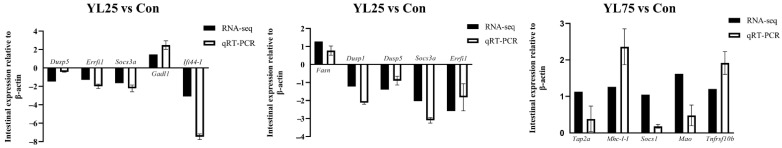
Gene relative expression in response to different dietary YL treatments as detected by qRT-PCR. Data were shown as means ± SD (standard deviation). The relative expression values were adjusted to the β-actin gene. Con was the control diet. In the other three diets, 25%, 50%, 75% of the fish meal was replaced with YL. The diets were named YL25, YL50, and YL75, respectively.

**Figure 8:**
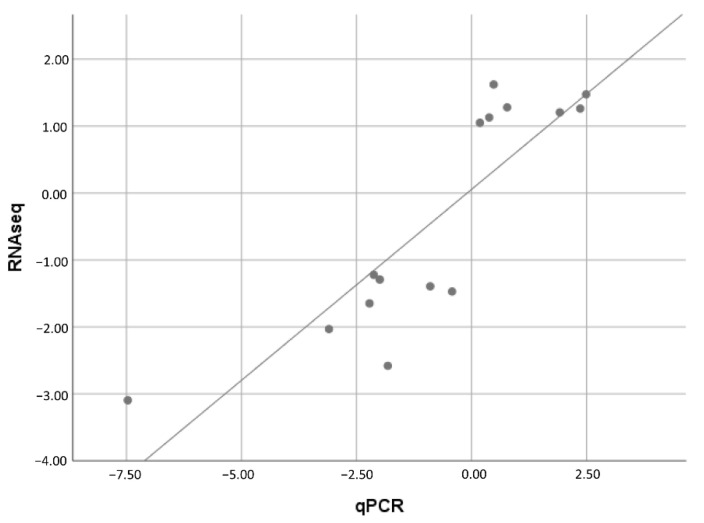
Validation of RNA-seq data with qRT-PCR. The expression data of genes detected by RNA-seq were plotted against those that were detected by qRT-PCR. The reference line indicates the linear correlation between the RNA-seq and qRT-PCR.

To further evaluate the RNA-seq result, the mRNAs of seven upregulated DEGs (*fasn*, *socs1*, *gadl1*, *mao*, *tnfrsf10b*, *tap2*, and *MHC-I-l*) and five downregulated DEGs *(dusp1, dusp5, errfil1, ifi44-l*, and *socs3a*) were measured by qRT-PCR. As shown in Figure 7, the expression data from qRT-PCR showed an expression tendency similar to those of RNA-seq for regulated genes, despite some quantitative differences at the expression level. The correlation between RNA-seq and qRT-PCR data was analyzed by the Spearman rho test and a highly correlated significance (*p* < 0.01, correlation coefficient = 0.868) was observed (Figure 8). The qRT-PCR analysis confirmed the expressions of DEGs detected by the high-throughput sequencing analysis.

## 3. Discussion

The improved growth performance induced by dietary YL inclusion have been confirmed in many animals, including piglets (*Sus scrofa*) [27], dairy calves (*Bos taurus*) [28], turkey (*Meleagris gallopavo*) [29], and Atlantic salmon (*Salmo salar* L.) [19]. It has been well demonstrated that the nucleic acid content in yeast is 50 g/kg to 100 g/kg, which is much higher than that of most animal and plant protein sources [30]. A previous study demonstrated that exogenous nuclei acid could act as a feed enhancer for fish. Kubitza et al. found an effective dietary concentration of inosine and inosine-5-monophosphate (2800 mg/kg) of feed, which provided a feed intake that was 23% higher than that of the control diet (*p* < 0.05) and improved the growth performance of juvenile largemouth bass [31]. Burrells et al. found that the growth benefits following feeding of Atlantic salmon with nucleotide-supplemented diets could be partly due to an increase in the mucosal surface area of the gut, due to a significantly enhanced intestinal fold morphology [32]. A diet containing 360 g/kg *Methylococcus capsulatus protein* (nucleic acids content, 88.2 g/kg) had no adverse effects on the appetite of Atlantic salmon, but significantly increased the FBW and SGR [33]. Therefore, higher nuclei acid content in a diet might play an important role in improving fish growth. 

After a 28 day feeding trial of Atlantic salmon, Berge et al. found that the weight and SGR was significantly higher for fish that were fed a diet containing 200 g/kg YL, compared with fish that were fed a fishmeal-based control diet (*p* < 0.05) [19]. Other trials with yeast-based proteins have yielded comparable results. Sunshine bass fed with an experimental diet (50% of the FM content was replaced with ethanol yeast) demonstrated significantly higher weight gain [34]. 

Consistently, a higher growth rate, without negative effects on HSI and CF, was observed in the YL25 (containing 150 g/kg YL) and YL50 (containing 300 g/kg YL) treatments, and the optimum dose of dietary YL required for maximum growth was 189.82 g/kg (with 31.63% of the fishmeal replaced by YL) according to the quadratic polynomial test and regression relationships, while dietary YL75 (containing 450 g/kg YL) remarkably decreased the SGR and CF. A previous report demonstrated that HSI often serves as an important parameter in assessing the nutritional status and physiological condition of fish [35], and CF is used to compare the body condition, fatness, or health of fish populations [36]. These results illustrated that a certain degree of YL substitution in FM could maintain proper physiological conditions, but excessive amounts of YL may be harmful to juvenile largemouth bass.

Furthermore, to determine the effect of dietary YL on fish growth at the gene level, an RNA-seq based transcriptome assembly was utilized and the mRNA expression levels were compared. A published report demonstrated that the ERBB receptor feedback inhibitor 1 (ERRFI1) inhibits the activity of the epidermal growth factor receptor (EGFR) catalytic by interfering with its dimerization [37]. The lower mRNA level of *errfi1* indicated that the inclusion of dietary YL25 and YL50 might maintain the EGFR activity at a high level and positively regulate fish growth. In addition, it has been confirmed that SOCS1 strongly inhibited the growth hormone receptor (GHR), and further decreased the growth of the host [38,39]. More recently, Zhao et al. established that SOCS1 could inhibit the effect of the growth hormone that played an important role in regulating the growth and development of blunt snout bream [40]. In the present study, dietary YL75 significantly promoted the expression of SOCS1, indicating that excessive YL content (75% substitution) in FM may negatively affect fish growth.

Our transcriptional analysis showed that certain genes associated with lipids’ metabolism were regulated by YL inclusion treatments. For instance, fatty acid synthase (FASN), a pivotal lipogenic enzyme catalyzing the terminal steps in the de novo biogenesis of fatty acids, is important for the adipose deposition of the host [41]. In fish, a previous report demonstrated that higher levels of FASN could stimulate the unsaturated fatty acid biosynthesis and lipid accumulation in the body [42]. The fatty acid-binding protein, intestinal (FABP2) is thought to play a role in the intracellular transport of long-chain fatty acids and their acyl-CoA esters, and is probably involved in triglyceride-rich lipoprotein synthesis [43,44]. Our data showed that dietary YL50 significantly upregulated the expression of FASN and FABP2, indicating that YL50 increased de novo lipogenesis, which might account for the highest lipid content in flesh. In addition, we found that dietary YL inclusion upregulated the mRNA level of AA metabolism-related genes, including acidic amino acid decarboxylase (*gadl1*) with YL25 treatment, glycine decarboxylase (*gldc*) and formimidoyltransferase-cyclodeaminase (*ftcd*) with YL50 treatment, and flavin containing amine oxidoreductase (*mao*) with YL75 treatment, which were all associated with the process of amino acids’ degradation or transamination [45,46,47,48]. This indicates that dietary YL might regulate AA metabolism to produce adequate protein sources for later growth process.

Microbiota analysis showed that Firmicutes were the dominant taxon in all four treatments, followed by Proteobacteria and Bacteroidetes at the phylum level (Figure 4). In addition, the Firmicutes/Bacteroidetes ratio was 2.065, 3.314, 7.345, and 2.012 in the Con, YL25, YL50, and YL75 treatments, respectively. A previous report demonstrated that the ratio of Firmicutes/Bacteroidetes reflected nutrient transportation and utilization [49,50], revealing that the dietary YL25 and YL50 inclusions might enhance the digestion and absorption of nutrients, thereby leading to a higher growth rate of juvenile largemouth bass. 

Moreover, our data revealed that Mycoplasma was the dominant taxon in the YL25 and YL50 treatments, the content of which decreased to a low level in the YL75 treatment at the genus level. Mycoplasma is a genus of comprises small, ubiquitous bacteria that lack a rigid cell wall [51], with great potential to cause intestinal inflammatory diseases in terrestrial animals [52]. Nevertheless, growing evidence shows that Mycoplasma is the main taxon existing in healthy largemouth bass intestine [8,9,22,53,54], while the role of *Mycoplasma sp.* in fisheries is not clear at present. Some insight into the potential role of Mycoplasma in fisheries revealed that the identified metabolism pathway encoding genes in Mycoplasma, such as riboflavin, are crucial for the fish growth and reproduction [55,56]. In this work, dietary YL25 and YL50 inclusion significantly increased Mycoplasma content, with a similar tendency in growth performance, indicating that Mycoplasma or its metabolites might also play a role in the growth of juvenile largemouth bass.

In contrast, the abundance of Lachnospiraceae decreased in the intestines of fish following the administration of dietary YL50. All members of Lachnospiraceae are anaerobic, fermentative, and chemoorganotrophic, and some display strong hydrolyzing activities [57]. A genomic analysis of Lachnospiraceae revealed a considerable capacity to utilize diet-derived polysaccharides, including starch and arabinoxylan, with substantial variability among species and strains [58]. Interestingly, the expression of amylase alpha 2A (*amy2a*) that is involved in carbohydrate metabolism was downregulated in the YL50 treatment. AMY2A is known to endo-hydrolyze (1, 4)-alpha-D-glucosidic linkages in polysaccharides [59]. Therefore, the downregulation of AMY2A and the decrease in Lachnospiraceae might indicate a modulation of the amylase activity by the YL50 inclusion in diet. Nevertheless, no negative effects of dietary YL50 on growth were observed in the present study, which might be attributable to the fact that dietary protein and lipids play a more important role for juvenile largemouth bass in providing the basis for growth, essential fatty acids, and metabolic energy than that of carbohydrates [60]. 

In aquatic animals, it has been confirmed that MAPK signaling is critically involved in the innate immune response to pathogen-associated molecular patterns (PAMPs) [61,62]. Meanwhile, dual-specificity phosphatases (DUSPs) have been proposed as a set of molecular control devices for specifying and modulating MAPK signaling, which may be targeted to unleash or attenuate innate and adaptive immune effector functions [63,64]. For instance, DUSP1 is a central negative regulator of innate immunity, receiving inputs from both pro- and anti-inflammatory stimuli and controlling responses by modulating the duration of JNK and p38 MAPK activation, whose inhibition could lead to promoting the production of inflammatory cytokine TNF-α and IL-1β [65,66]. Similarly, DUSP5-deficient mice exhibited increased cellular ERK1/2, IL-33-mediated activation, and pro-inflammatory cytokines secretion (IL-1β, IL-6, and TNF-α), which could act as negative feedback regulators of the MAPK pathway and which are critical for normal immune system development [67,68,69]. Conversely, DUSP2 could inactivate ERK1/2 and p38MAPK, and has been confirmed to positively regulate the innate immune response [70]. In fish, previous report has also evidenced that DUSP1, DUSP2, and DUSP5 *5* play an important role in regulating the MAPK-dependent immune responses in the Japanese flounder [62]. In the present study, compared with control treatment, DUSP1, DUSP2, and DUSP5 were remarkably downregulated in the YL25 and YL50 treatments. These results indicated that the opposite effects of DUSPs (DUSP2 vs. DUSP1/5) may provide an underlying mechanism by which the MAPK-dependent inflammatory responses are properly balanced and thus maintain an appropriate immune defense in fish with YL25 and YL50 treatments.

Antioxidant enzyme activities are the main cytoprotective mechanisms against oxidative stress in fish tissues [71]. A previous study demonstrated that aquatic animals fed with *Y. lipolytica* (1.1% of the basal diet) showed higher antioxidant enzyme activities [20,21] than other animals. Moreover, the incubation of leukocytes (head, kidney and spleen) with *Y. lipolytica* increased the leucocyte functions of Pacific red snapper, such as phagocytic ability, antimicrobial activity, respiratory burst activity, and SOD activity [72]. These results indicated the potential application of a *Y. lipolytica*-based immunostimulant for aquatic animals. 

In this work, the T-AOC, SOD, and GSH-Px activity showed a tendency to decrease, after an initial increase, with the increase in the YL inclusion level in a quadratic manner. The lowest activity of these critical antioxidant enzymes was determined in the YL75 treatment. In addition, shorter and sparse intestinal villi with a distorted crypt structure were observed. These results indicated that a certain degree of YL substitution in FM had no negative effect on antioxidant capacity or intestine health, while a high level YL in diets may damage the intestines of juvenile largemouth bass. Moreover, our microbiota analysis showed that *Enterobacteriaceae* content increased significantly with YL75 treatment. It was confirmed that *Enterobacteriaceae*, including representatives of *Edwardsiella* and *Yersinia*, may induce intestinal colitis and facilitate immunopathologic responses in fish [73,74]. In this study, the expressions of the transporter associated with antigen processing 2, subunit type a(*tap2a*), and the major histocompatibility complex class I-related gene (*MHC1-l*), which is involved in phagosome and cell adhesion molecules, were significantly upregulated with YL75 treatment. TAP2a plays a key role in the adaptive immune response by pumping antigenic peptides into the endoplasmic reticulum for subsequent loading of MHC class I molecules [75]. MHC presents microbial antigens to recognize diverse microbes and to elicit rapid innate-type cell activation to eliminate pathogenic microbes by directly killing infected cells [76]. In fish, a published report confirmed the MHC class I molecules of Japanese flounder [77]. Meanwhile, growing evidence showed that the expression of MHC I was upregulated by pathogens’ infections [78,79,80]. For example, treatment of blunt snout bream with *Aeromonas hydrophila* resulted in a remarkable increase in the expression of *MHC I*, within 72 h after infection in the gills, kidneys, and intestines [78]. In our study, the significantly elicited expression of the *tap2a* and *MHC1-l* genes in the YL75 treatment was likely due to the dramatic changes in microbes, which triggered an imbalance in immune homeostasis, and the increased contact between the expanding *Enterobacteriaceae* and the intestinal epithelium, which induced the expression of the *tap2a* and *MHC1-l*.

## 4. Materials and Methods

### 4.1. Autolyzed Yarrowia lipolytica Preparation

Marine *Yarrowia lipolytica* (YL) was kindly provided by Dr. Jinyong Yan (College of Life Science & Technology, Huazhong University of Science and Technology, China) [81]. The YL was cultured in a 300 mL YPD medium (10 g L^−1^ yeast extract, 20 g L^−1^ peptone, 50 g L^−1^ sucrose) using a 3000 mL conical flask with a 2% inoculation amount for 48 h at 28 °C with 220 rpm. After centrifugation at 5000 g for 30 min, the cell precipitation was collected and washed twice with sterile water, then autolyzed by incubating at 50 °C for 24 h in a bioreactor system (Bai lun Biotech Co., Ltd., Shanghai, China), with constant stirring at 50 rpm using a helical impeller. The autolysis was followed by freeze-drying (SCIENTZ-10N/A, Scientzbio, Ningbo, China) to prepare an autolyzed YL dry powder. In addition, the amino acid (AA) composition of YL and FM was assayed by the Pony Testing International Group (Shanghai, China), as shown in Table S5.

### 4.2. Experimental Diets

Four diets were formulated with graded substitution of YL in FM: 0% (control, YL0), 25% (YL25), 50% (YL50), and 75% (YL75). All ingredients were ground through a 200 μm mesh and weighed according to the formulation, and then mixed with the fish oil and water via a blending machine. Subsequently, pellets were prepared using a commercial pelletizer (KCHL-10, Kcth Group, Beijing, China). Then, the experimental diets were produced and dried in a cool and dry place away from light and stored at −20 °C for further use. The ingredients and proximate composition of the experimental diets are shown in Table 4.

### 4.3. Experimental Design

Two-month-old juvenile largemouth bass (“ZheLu” No.1) were purchased from a commercial farm (Huzhou Baijiayu Biotech Co., Ltd., Zhejiang, China). The fish were cultured in a circulating plastic tank that was set to flow in 200 L/h water throughout the experiment. The water temperature, pH, and dissolved oxygen were controlled at 25.0 ± 1.0 °C, 7.2–7.5, and 5.0 mg/L, respectively. The juvenile largemouth bass were acclimated to farming conditions by a commercial diet (Haida Co., Ltd., Guangzhou, China) for 7 days prior to the experiment.

A total of 600 juvenile largemouth bass, with an average body weight of approximately 1.07 g, were randomly divided into four treatments of 50 fish per tank with three replicates in each treatment, then weighed to obtain the initial body weights. Four diets were used in the feeding trial, and each experimental diet was fed to the fish thrice daily (08:00, 12:00, and 16:00) until apparent satiation. The feeding trial continued for 28 days in the conditions described above.

### 4.4. Sampling

The body weight and body length of the largemouth bass were measured at the beginning and at the termination of the feeding trail. Before sampling, all of the juvenile largemouth bass were fasted for 24 h, then anesthetized with tricaine methanesulfonate (50 mg L^−1^ water). All of the fish were collected and sacrificed to measure the individual body weights and lengths and to calculate for weight gain rate (WGR), hepatosomatic index (HSI), and condition factor (CF). Then, 24 fish per replicate were used for the transcriptome, microbiome, and qRT-PCR analysis. Eighteen fish per replicate were dissected to collect flesh and intestines for the analysis of antioxidant-related enzyme activity. Another three fish per replicate were dissected for the intestinal histological examination.

The WGR was calculated according to Equation (1), and the SGR, FCR, CF, and HSI were calculated according to Equations (2), (3), (4), and (5), respectively.

WGR (%) = 100 × (FBW (g) − IBW (g))/IBW (g)(1)

SGR (%/d) = 100 × [ln FBW (g) − ln IBW (g)]/28(2)

FCR (%) = I/(Wt − W_0_)/W_2_ (3)

CF (g/cm^3^) = 100 × (body weight/body length^3^) (4)

HSI (%) = 100 × (liver wet weight/whole wet weight)(5)

where IBW (g) was the initial body weight; FBW (g) was the final body weight; I (g) was the total amount of the test diets consumed by the fish; W_0_ (g) was the total initial body weight; and W_t_ (g) was the total final body weight.

### 4.5. Analysis of Flesh Composition

The proximate composition of the diets and the fish flesh were analyzed using the AOAC method (AOAC, 2000). The moisture of the collected samples was measured by drying to a constant weight at 105 °C. Crude protein was calculated from the determination of the total nitrogen (N × 6.25), using the Kjeldahl method (2300-Autoanalyzer, FOSS, Hilleroed, Denmark), after acid digestion, distillation, and titration. Crude lipid was determined by gravimetric analysis, following ether extraction of the lipids according to the Soxhlet method (36680-analyzer, BUCHI, Flawil, Switzerland). Ash was determined by incineration in a muffle furnace at 550 °C for 8 h [22].

### 4.6. Analysis of Antioxidant-Related Enzyme Activities in Intestine

To analyze the antioxidant capacity of juvenile largemouth bass fed with the four experimental diets, the total antioxidant capacity (T-AOC), superoxide dismutase (SOD), and glutathione peroxidase (GSH-Px) in the intestines were measured using commercially available assay kits (Nanjing Jiancheng Bioengineering Institute, Nanjing, China).

### 4.7. Analysis of Intestinal Histology

The sampled fish were dissected to obtain the same sections of midgut for each sample. After immersion in 4% Bouin’s solution for 24 h, all samples were dehydrated with the standard protocols and embedded in paraffin, with subsequent sectioning at 4 μm thicknesses [82]. Tissues sections were stained according to the protocols of hematoxylin-eosin (H&E) staining and subsequently examined under a light microscope (Leica DM2500, Leica, Wetzlar, Germany), using Suite software after the pictures were taken and prior to calibration. This was done by randomly choosing three villi from each sample (*n* = 3), The average of each replicate was used for statistical analysis.

### 4.8. RNA Extraction, cDNA Library Construction, Sequencing, De Novo Assembly and Annotation

Total RNA was extracted from the intestines of the fish for cDNA library construction, as previous described [83,84]. Then, the qualified library was pooled, based on a pre-designed target data volume, and sequenced on an Illumina sequencing platform at Beijing Biomarker Technologies Co., Ltd. (Beijing, China). De novo assembly and annotation were manipulated according to our previous study [85]. Briefly, the Q20, Q30, GC-content, and sequence duplication levels of the clean data were calculated. Genes were annotated by querying against GO and KEGG databases, using BLASTx [86,87].

### 4.9. Differential Expression Analysis and Functional Enrichment

The expression level of each transcript was calculated according to the reads per kilobase of the exon-per-million mapped reads (RPKM) method [4]. Then, the differentially expressed genes (DEGs) in the control and dietary YL treatments were identified using the DESeq2. Genes with expression fold change ≥ 2 and FDR < 0.01, found by DESeq2, were assigned as differentially expressed genes(Tables S6–S8). Moreover, GO enrichment analysis was carried out with GOATOOLS (https://github.com/tanghaibao/Goatools, accessed on 14 February 2022) for analysis of the DEGs. A KEGG pathway enrichment analysis was operated with KOBAS (http://kobas.cbi.pku.edu.cn/home.do, accessed on 14 February 2022) at a Bonferroni-corrected *p*-value ≤ 0.05, compared with the whole-transcriptome background. 

### 4.10. Quantitative Real-Time PCR

To verify the reliability of the DEG data, twelve representative DEGs (*Fasn*, *Dusp1*, *Dusp5*, *Errfi1*, *Tap2a*, *MHC*, *Socs1l*, *Mao*, *Socs3a*, *Gadl1*, *IfI44l*, and *Tnfrsf10b*) were chosen for qRT-PCR analysis. Briefly, RNA was extracted from the collected intestines with a TRIzol reagent (Tiangen, China), then synthesized with cDNA after a quality evaluation using a Nanodrop 2000. Subsequently, the cDNA was adjusted 100 ng/μL, and qRT-PCR was performed with a SYBR Green I Master Mix (Tiangen, China) in a LightCycler^®^ 480 II real-time system (Roche, Switzerland). Each assay was conducted in triplicate with the β-actin gene as the internal reference and analyzed relative to the β-actin gene by the 2^−ΔΔCt^ method [88,89]. All primers for the qRT-PCR were designed based on specific sequences, as listed in Table S9.

### 4.11. High Throughput Sequencing of the 16S rRNA Gene

The intestinal contents were collected from the whole intestines of juvenile fish in four groups for high-throughput sequencing of the 16S rRNA gene. The microbial DNA was extracted using a E.Z.N.A.^®^ Soil DNA kit (Omega Biotek, Norcross, GA, U.S.). The V3-V4 region of the bacterial 16S ribosomal RNA gene was amplified by PCR using the primers 338F 5′-ACTCCTACGGGAGGCAGCAG-3′ and 806R 5-GGACTACHVGG-TWTTAAT-3′. Then, amplicon DNA was extracted and purified, and SMRTbell libraries were prepared by blunt-end ligation according to the manufacturer’s instructions (Pacific Biosciences). Subsequently, purified SMRTbell libraries were sequenced by Beijing Biomarker Technologies Co., Ltd. (Beijing, China).

According to the quality of a single nucleotide, raw data were primarily filtered by Trimmomatic [90] (version 0.33). The identification and removal of primer sequences were processed by Cutadapt [91] (version 1.9.1). PE reads obtained from previous steps were assembled by USEARCH [92] (version 10) and followed by chimera removal using UCHIME [93] (version 8.1). The high-quality reads generated from the above steps were used in the following analysis. Then, OTU clustering, species annotation, alpha/beta diversity, microbial community bar plots, heat maps, phylogenetic analyses, principal component analysis (PCA), and linear discriminant analysis effect size (LefSe) were performed using BMKCloud (www.biocloud.net, accessed on 14 February 2022).

### 4.12. Statistics Analysis

The experimental data were presented with SPSS version 20.0 software (Version 20.0; SPSS, Inc., Chicago, IL, USA). Polynomial contrasts were used to determine the linear and quadratic effects of dietary YL inclusion levels for the variables that were measured. The level of significance was set at *p* < 0.05, and the results were presented as mean values, plus or minus standard errors. The intestinal bacterial diversity and the relative expression of genes were analyzed by one-way analysis of variance (ANOVA), followed by Duncan’s post hoc test, with statistically significance being considered as *p* < 0.05.

## 5. Conclusions

Our complementary analysis of the intestinal transcriptomic profiling and microbiota response to dietary YL aimed to take a further step in the evaluation of FM. YL can replace up to 50% of dietary FM to promote growth performance without any adverse influence on the intestinal health of juvenile largemouth bass. Nevertheless, the high inclusion of dietary YL would have significant negative effects on the growth performance and intestinal health of the fish. Although our study was a short-term screening trial, our results indicated, at this stage, that YL is a feasible FM substitute, at 189.82 g/kg, in the diet of juvenile largemouth bass.

## Figures and Tables

**Table 1:** Growth performance and feed utilization of largemouth bass fed the experimental diets for 28 days *.

	Diets				Pooled SEM	Regression (P/R^2^)
	Con	YL25	YL50	YL75		Quadratic
IBW (g)	1.07	1.07	1.06	1.11	n.a.	n.a.
FBW (g)	4.52	4.77	4.85	4.13	0.09	0.006/0.678
WGR (%)	322.43	345.79	357.55	272.07	8.55	<0.001/0.817
SGR (%/day)	5.15	5.34	5.43	4.69	0.08	<0.001/0.818
FCR	1.06	1.05	1.02	1.31	0.03	0.001/0.778
CF (g/cm^3^)	1.40	1.43	1.49	1.32	0.04	0.007/0.668
HSI (%)	1.79	1.82	1.90	1.59	0.12	n.s.

* Values are means of triplicate. SEM, standard error of means; n.a., not applicable; n.s., no significant difference was observed; IBW, initial body weight; FBW, final body weight; WGR, weight gain rate; SGR, specific growth rate; FCR, feed conversion ratio; HSI, hepatosomatic index; CF, condition factor. Con was the control diet. In the other three diets, 25%, 50%, and 75% of the fish meal was replaced with YL. The diets were named, respectively, YL25, YL50, and YL75.

**Table 2:** Effects of dietary YL on flesh composition of juvenile largemouth bass fed the experimental diets for 28 days *.

	Diets				Pooled SEM	Regression (P/R^2^)
	Con	YL25	YL50	YL75		Quadratic
Moisture (%)	74.86	74.91	74.55	74.87	0.24	n.s.
Crude protein (%)	17.89	17.95	18.02	17.81	0.17	n.s.
Crude lipid (%)	3.47	3.97	4.33	3.19	0.06	<0.001/0.949

* Values are means of triplicate. SEM, standard error of means; n.s., no significant difference was observed. Con was the control diet. In the other three diets, 25%, 50%, 75% of the fish meal was replaced with YL. The three diets were named, respectively, YL25, YL50, and YL75.

**Table 3:** Histological parameters of mid-intestinal in juvenile largemouth bass fed different YL inclusion diets *.

	Diets				Pooled SEM	Regression (P/R2)
	Con	YL25	YL50	YL75		Quadratic
Villus height (μm)	386.38	434.22	449.18	312.86	15.92	0.002/0.760
Villus width (μm)	59.83	57.12	62.03	49.65	4.70	n.s

* Values are means of triplicate. SEM, standard error of means; n.s., no significant difference was observed. Con was the control diet. In the other three diets, 25%, 50%, 75% of the fish meal was replaced with YL. The diets were named, respectively, YL25, YL50, and YL75.

**Table 4:** Ingredients and proximate composition of the experimental diets ^1^.

Ingredients (g/kg)	Diets			
	Con	YL25	YL50	YL75
Fish meal	600	450	300	150
Fermented soybean meal	120	120	120	120
Shrimp meal	50	50	50	50
YL	0	150	300	450
Fish oil	50	50	50	50
Wheat flour	50	50	50	50
Gluten	40	40	40	40
Soluble starch	40	40	40	40
Microcrystalline fiber	10	10	10	10
Ca(H_2_PO_4_)2	10	10	10	10
Mineral premix 2	12	12	12	12
Vitamin premix 3	10	10	10	10
Choline chloride	3	3	3	3
L-lysine	3	3	3	3
L-methionine	2	2	2	2
Proximate composition (%)				
Dry matter (%)	93.42	93.75	93.93	94.06
Crude protein (%)	50.34	50.07	49.83	49.75
Crude lipid (%)	9.42	9.51	9.56	9.63
Ash (%)	10.45	10.57	10.61	10.65

^1^ Con was the control diet. In the other three diets, 25%, 50%, and 75% of the fish meal was replaced with YL and the diets were named as YL25, YL50, and YL75, respectively. ^2^ Mineral premix (mg/kg diet): CuSO₄·5H₂O, 10; FeSO_4_·H2O, 300; ZnSO_4_·H_2_O, 200; MnSO_4_·H2O, 100; KIO_3_ (10%), 80; Na_2_SeO_3_ (10% Se), 67; CoCl_2_·6H_2_O (10% Co) 5; NaCl, 100; zeolite, 638. ^3^ Vitamin premix (mg/kg diet): vitamin A, 20; vitamin B1, 12; vitamin B2, 10; vitamin B6, 15; vitamin B12 (1%), 8; vitamin C, 200; niacinamide, 100; ascorbic acid, 400; calcium pantothenate, 40; biotin (2%), 2; folic acid, 10; vitamin E, 200; vitamin K3, 10; vitamin D3, 10; inositol, 200.

## Data Availability

All 16s amplicon sequencing data and transcriptional sequencing data have been deposited in NCBI under accession number PRJNA857532. All other datasets generated for this study are included in the article. The data that support the findings of this study but not presented in the figures, tables, and supplementary files are available upon reasonable request.

## Supplementary Materials

The following supporting information can be downloaded at: https://www.mdpi.com/article/10.3390/ijms231810780/s1.
