# Supplementary material for: Effect of Autolyzed Yarrowia lipolytica on the Growth Performance, Antioxidant Capacity, Intestinal Histology, Microbiota, and Transcriptome Profile of Juvenile Largemouth Bass (Micropterus salmoides)
Source: Int J Mol Sci. 2022 Sep 15;23(18):10780. doi: 10.3390/ijms231810780 (PMC9503160; doi:10.3390/ijms231810780)
Supplement: Supplementary file 1 [file ijms-23-10780-s001.zip › Table S1.pdf]

Table S1. Raw reads and clean reads of 16 sRNA sequence

Sample ID	Raw Reads	Clean Reads	Denoised Reads	Merged Reads	Non-chimeric Reads
conI	80256	80160	79918	75345	72724
conII	80056	79963	79776	76216	74187
conIII	79979	79881	79500	72251	70275
YL25I	79879	79793	79438	73171	70911
YL25II	79905	79783	79217	69142	66561
YL25III	79915	79800	79587	75273	73942
YL50I	79916	79838	79657	76518	75378
YL50II	80053	79946	79586	75089	73309
YL50III	80160	80051	79821	76523	75532
YL75I	79634	79531	79361	75368	72654
YL75II	80178	80073	79766	76578	73849
YL75III	80031	79920	79614	75020	72936

Abbreviations: Con was the control diet. In the other 3 diets, 25%, 50%, 75% of the fish meal in the diet was replaced with YL, named as YL25, YL50, and YL75, respectively.
