# Supplementary material for: Effect of Autolyzed Yarrowia lipolytica on the Growth Performance, Antioxidant Capacity, Intestinal Histology, Microbiota, and Transcriptome Profile of Juvenile Largemouth Bass (Micropterus salmoides)
Source: Int J Mol Sci. 2022 Sep 15;23(18):10780. doi: 10.3390/ijms231810780 (PMC9503160; doi:10.3390/ijms231810780)
Supplement: Supplementary file 1 [file ijms-23-10780-s001.zip › Table S2.pdf]

Table S2. Alpha diversity of intestinal microbiota of largemouth bass fed the experimental diets for 28 days*.

Item	Con	YL25	YL50	YL75
**ACE**	1898.93±489.29	1984.06±513.18	1300.84±271.40	1461.80±247.68
**Shannon**	9.92±0.22[a]	8.33±1.27[ab]	6.36±1.78[b]	9.72±0.02[a]
**Simpon**	1.00±0.00[a]	0.94±0.06[ab]	0.82±0.12[b]	1.00±0.00[a]
**Chao1**	1897.92±490.04	1983.52±513.08	1300.20±271.32	1459.93±248.04

Abbreviations: Con was the control diet. In the other 3 diets, 25%, 50%, 75% of the fish meal in the diet was replaced with YL, named as YL25, YL50, and YL75, respectively. Bars assigned with different letters were significantly different ($p < 0.05$)
