# Supplementary material for: Effect of Autolyzed Yarrowia lipolytica on the Growth Performance, Antioxidant Capacity, Intestinal Histology, Microbiota, and Transcriptome Profile of Juvenile Largemouth Bass (Micropterus salmoides)
Source: Int J Mol Sci. 2022 Sep 15;23(18):10780. doi: 10.3390/ijms231810780 (PMC9503160; doi:10.3390/ijms231810780)
Supplement: Supplementary file 1 [file ijms-23-10780-s001.zip › Table S3.pdf]

Table S3. Reads and GC content of transcriptome sequence.

Samples	Clean reads	Clean bases	GC Content	%≥Q30
conI	20,091,039	6,004,089,864	47.04%	96.21%
conII	21,644,379	6,456,408,806	48.40%	97.02%
conIII	21,146,017	6,320,248,636	48.63%	96.80%
YL25I	20,646,689	6,169,456,966	47.63%	96.71%
YL25II	20,517,227	6,123,797,612	48.67%	96.69%
YL25III	21,209,558	6,338,291,336	48.05%	96.70%
YL50I	20,079,215	6,003,054,836	48.49%	96.72%
YL50II	21,993,661	6,566,425,000	48.63%	96.70%
YL50III	21,197,344	6,334,653,974	46.83%	96.29%
YL75I	20,843,766	6,230,819,350	48.23%	96.70%
YL75II	20,945,091	6,256,602,202	47.38%	96.80%
YL75III	21,355,472	6,383,318,998	47.62%	97.00%

Abbreviations: Con was the control diet. In the other 3 diets, 25%, 50%, 75% of the fish meal in the diet was replaced with YL, named as YL25, YL50, and YL75, respectively.
