# Supplementary material for: Effect of Autolyzed Yarrowia lipolytica on the Growth Performance, Antioxidant Capacity, Intestinal Histology, Microbiota, and Transcriptome Profile of Juvenile Largemouth Bass (Micropterus salmoides)
Source: Int J Mol Sci. 2022 Sep 15;23(18):10780. doi: 10.3390/ijms231810780 (PMC9503160; doi:10.3390/ijms231810780)
Supplement: Supplementary file 1 [file ijms-23-10780-s001.zip › Table S4.pdf]

Table S4. Partial of DEGs involved in growth, metabolism and immunity

| Treatment | Acronym | Gene name | Log2FC | Regulated | FDR |
|---|---|---|---|---|---|
| YL25 | *Errfi1* | *ERBB receptor feedback inhibitor 1* | 1.292360 | down | 0.0072430 |
| | *Gadl1* | *Acidic amino acid decarboxylase GADL1* | 1.474174 | up | 0.0077045 |
| | *Dusp5* | *dual specificity phosphatase5* | 1.471459 | down | 2.52E-05 |
| YL50 | *Errfi1* | *ERBB receptor feedback inhibitor 1* | 2.581062 | down | 5.17E-10 |
| | *Fasn* | *fatty acid synthase* | 1.278785 | up | 0.0003678 |
| | *Fabp2* | *fatty acid-binding protein, intestinal* | 1.508187 | up | 0.000166486 |
| | *Ftcd* | *formimidoyltransferase-cyclodeaminase* | 1.117450 | up | 0.0003119 |
| | *Gldc* | *glycine decarboxylase* | 1.247167 | up | 0.0077539 |
| | *Amy2a* | *amylase alpha 2A* | 2.576246 | down | 6.99E-11 |
| | *Dusp1* | *dual specificity phosphatase1* | 1.222461 | down | 0.0025691 |
| | *Dusp5* | *dual specificity phosphatase5* | 1.393692 | down | 0.0002042 |
| YL75 | *Socs1* | *suppressor of cytokine signaling 1* | 1.049256 | up | 0.0054801 |
| | *MAO* | *flavin containing amine oxidoreductase* | 1.620982 | up | 0.0003471 |
| | *Tap2a* | *transporter associated with antigen processing 2, subunit type a* | 1.128581 | up | 1.399E-07 |
| | *MHC-I-l* | *major histocompatibility complex class I-related gene protein-like* | 1.262056 | up | 0.0026493 |
| | *Tnfrsf10b* | *tumor necrosis factor receptor superfamily member 10B* | 1.204773 | down | 0.0097271 |

**Abbreviations:** 25%, 50%, 75% of the fish meal in the diet was replaced with YL, named as YL25, YL50, and YL75, respectively.
