# Supplementary material for: Effect of Autolyzed Yarrowia lipolytica on the Growth Performance, Antioxidant Capacity, Intestinal Histology, Microbiota, and Transcriptome Profile of Juvenile Largemouth Bass (Micropterus salmoides)
Source: Int J Mol Sci. 2022 Sep 15;23(18):10780. doi: 10.3390/ijms231810780 (PMC9503160; doi:10.3390/ijms231810780)
Supplement: Supplementary file 1 [file ijms-23-10780-s001.zip › Table S5.pdf]

Table S5. Amino acid composition of the fish meal and Lyophilized *Yarrowia lipolytica* (dry matter basis，%）

Amino acid	Fish meal	Lyophilized *Yarrowia lipolytica*
Asp	4.64	3.95
Thr	3.10	3.03
Ser	6.06	2.60
Glu	7.65	4.76
Pro	5.56	2.66
Gly	5.84	2.78
Ala	3.53	4.64
Cys-Cys	0.70	0.56
Val	4.07	2.97
Met	0.87	2.10
Ile	2.94	2.60
Leu	5.39	3.89
Tyr	2.24	1.73
Phe	3.11	2.34
Lys	3.69	4.14
His	0.87	1.49
Arg	4.60	2.22
Trp	/	0.56
