# Supplementary material for: Effect of Autolyzed Yarrowia lipolytica on the Growth Performance, Antioxidant Capacity, Intestinal Histology, Microbiota, and Transcriptome Profile of Juvenile Largemouth Bass (Micropterus salmoides)
Source: Int J Mol Sci. 2022 Sep 15;23(18):10780. doi: 10.3390/ijms231810780 (PMC9503160; doi:10.3390/ijms231810780)
Supplement: Supplementary file 1 [file ijms-23-10780-s001.zip › Table S9.pdf]

Table S9. Primers used in quantitative real-time PCR

Gene	Primers sequence(5' – 3')	Target size(bp)	Accession number
β-actin	F: TGGAAGGGACCTCACAGACTAC<br>R: GGGCAACGGAACCTCTCAT	231	MH018565
Fasn	F: GGCAGCATACACACAGACCT<br>R: GTGGTATTTGGTCGCAGGGA	289	XM_038735140.1
Dusp1	F: CATATCGGGCTCCACCAACG<br>R: AAAACGTTTCAAAACCGCCTCTG	269	XM_038700310.1
Dusp5	F: GGCCTTGCTCAATGTGTCG<br>R: ATGTCGAAGGCCGCATCTA	258	XM_038713959.1
Errfi1	F: GGCTTCTCTGGATTTTCATGTCTC<br>R: AGTGGAAAAGTGGACAGTGTGA	111	XM_038722921.1
Tap2a	F: GTGCAGGACTCAATGGCTCT<br>R: CAAACCTGTCAACCTGCGTG	195	XM_038725548.1
Mhc	F: GAAGATCAGAACCAACGAAGAGTC<br>R: TCAAACTTCTGTGAGGACAAATACC	100	XM_038694736.1
Socs1l	F: GGAGGCTACAAACTTGAACCG<br>R: GAAATGTCTGGTGAGGTGCAAT	144	XM_038712981.1
Mao	F: TAATACAGATGCGCCCCTCG<br>R: TCGCTGCACTCAAACCTGATA	150	XM_038721394.1
Socs3a	F: TTGGAGAAACGTTGAAGGCTGC<br>R: GCCCCAGTAGAAACCACTCT	202	XM_038701477.1
Gadl1	F: CACAGCTGAGAAATGGCCTCA<br>R: GAACGCCACTCACAGACCTTTT	254	XM_038712267.1
Ifl44l	F: GTCCAAGGCTCATATTCAGTCTTTC<br>R: GACTTTCCAGCTCCAACTGAT	236	XM_038727646.1
Tnfrsf10b	F: GGGGTTGGATGTAAGTGGTG<br>R: TGGAAAGGAAGGCAAGTGTC	150	XM_038696919.1
